# Prevalence and factors related to malocclusion, normative and perceived orthodontic treatment need among children and adolescents in Bangladesh

**DOI:** 10.1590/2177-6709.24.3.44.e1-9.onl

**Published:** 2019

**Authors:** Sharmin Sultana, Zakir Hossain

**Affiliations:** 1 Universiti Sains Malaysia, School of Dental Sciences (Kelantan, Malaysia).; 2 Dhaka Dental College & Hospital, Department of Orthodontics &Dentofacial Orthopedics (Dhaka, Bangladesh).

**Keywords:** Malocclusion, IOTN, Normative need, Perceived need, Schoolchildren

## Abstract

**Objective:**

The purpose of the present study was to assess the prevalence of normative and perceived orthodontic treatment need in schoolchildren and adolescents, related risk factors, and children/parent’s aesthetic perception, compared to orthodontist’s opinion, in Dhaka city, Bangladesh.

**Methods:**

A random sample of 800 schoolchildren aging 11-15 years was selected from different schools in the city of Dhaka, Bangladesh. The Dental Health Component (DHC) and Aesthetic Component (AC) of the Index of Orthodontic Treatment Need (IOTN) were assessed as normative treatment need. The Decayed, Missing, Filled Teeth (DMFT) index was used to record caries experience. Children were interviewed on the perception of orthodontic treatment need. Parents also completed a questionnaire on the perception of their child’s orthodontic treatment need, assessed by AC/ IOTN.

**Results:**

According to the DHC/IOTN, only 24.7% were in the category of definite need (grade 4-5) for orthodontic treatment. A significant difference was found between the clinician/children and clinician/parents perceived AC score of IOTN (*p*= 0.0001). Multiple logistic regression showed children with a higher DMFT were significantly more likely to need orthodontic treatment, according to the DHC of IOTN.

**Conclusion:**

A low proportion of schoolchildren needs normative orthodontic treatment in the city of Dhaka, Bangladesh. Children with a higher DMFT score were significantly more likely to need orthodontic treatment, according to the DHC of IOTN.

## RESUMO

**Objetivo:**

o objetivo do presente estudo foi avaliar a prevalência de necessidade real e percebida de tratamento ortodôntico em crianças e adolescentes em idade escolar, bem como de fatores de risco relacionados, e comparar a percepção estética das crianças e responsáveis com a percepção de ortodontistas na cidade de Dhaka, Bangladesh.

**Métodos:**

uma amostra aleatória composta por 800 crianças, com idades entre 11 e 15 anos, foi selecionada em diferentes escolas da cidade de Dhaka, Bangladesh. Para avaliação da necessidade real de tratamento, utilizou-se o *Dental Health Component* (DHC) e o *Aesthetic Component* (AC) do *Index of Orthodontic Treatment Need* (IOTN). O índice de Dentes Cariados, Perdidos e Obturados (CPO-D) foi usado para registrar a prevalência de cáries dentárias. As crianças foram entrevistadas sobre sua percepção da necessidade de tratamento ortodôntico, e os pais também responderam a um questionário sobre a percepção da necessidade de tratamento ortodôntico de seus filhos, avaliada pelo AC do IOTN.

**Resultados:**

de acordo com o DHC, apenas 24,7% das crianças foram alocadas na categoria de necessidade real (notas 4-5) de tratamento ortodôntico. As pontuações no AC foram significativamente diferentes entre os ortodontistas, as crianças e os responsáveis (*p*= 0,0001). A análise da regressão múltipla mostrou que as crianças com maiores pontuações no CPO-D tinham chance significativamente maior de precisar de tratamento ortodôntico, segundo o DHC.

**Conclusão:**

um número reduzido de crianças em idade escolar necessita realmente de tratamento ortodôntico em Dakha, Bangladesh. Além disso, as crianças com maior CPO-D apresentaram chance significativamente maior de precisar de tratamento ortodôntico, segundo o DHC.

## INTRODUCTION

Malocclusion may play an important role in social acceptance and interactions, for esthetic reasons, and may also result in functional limitations in more serious instances. Currently, much attention has been focused on assessing the severity and prevalence of malocclusion and orthodontic treatment need worldwide.^1^

Several local factors, such as adverse oral habits, tooth anomalies, shape and position of developing teeth may cause malocclusion. Children and adolescents who have misaligned teeth may feel embarrassed in social contacts, and may become deliberately irritated by their dental appearance.^1^ ^,^ ^2^ The spectrum of factors involves their self-respect and self-confidence, which largely depend on proper physical appearance, well-proportioned facial appearance, properly aligned teeth and a pleasing, attractive smile. The expanded approach in orthodontic treatment demands better evaluation of treatment needs and standardized criteria for their assessment. Indeed, it has been reported that a significant number of children are inappropriately referred for orthodontic treatment, underlying the necessity of objective or normative assessment of orthodontic treatment by using an index.^1^ ^,^ ^3^ ^-^ ^6^

The Index of Orthodontic Treatment Need (IOTN) is one of the most popular occlusal indices in Europe, and is gaining widespread use worldwide.^7^ The IOTN has two measuring components: the Aesthetic Component (AC) and the Dental Health Component (DHC). The DHC and the AC components of the IOTN have been used to assess the normative need.^8^ The AC assesses the perception of an individual on the attractiveness of his/her dentition, by means of a 10-point photographic scale, showing different levels of dental attractiveness. The DHC has five grades based on the worst occlusal trait, and each given grade is a reflection of the level of normative orthodontic treatment need, to support the treatment prioritization.^1^ ^,^ ^6^ ^-^ ^10^ Numerous studies have been published regarding the prevalence of malocclusion in various populations. The outcomes have demonstrated wide variations, with the reported prevalence ranging from 39 to 98%.^3^ Several studies have shown self-perception of malocclusion and need of orthodontic treatment for different populations, based on IOTN.^1^ ^,^ ^11^ However, the outcomes demonstrated that the need for orthodontic treatment was about one third to half of the children and adolescent population. Some previous studies have shown significant differences between children’s, parent’s and clinician’s perception of orthodontic treatment need.^1^ ^,^ ^5^ ^,^ ^6^ ^,^ ^12^ ^,^ ^13^ Few studies have considered parent’s perceptions of orthodontic treatment need^6^ ^,^ ^7^. Ultimately, parents take the final decision about their child’s treatment, and may have different motives for treatment than their children. It has also been reported that parents are the most influential in the motivation for treatment.^6^ ^,^ ^7^

In Bangladesh, a previous study assessed the need for orthodontic treatment among Bangladeshi schoolchildren in late mixed dentition stage, based on IOTN^14^. Meanwhile, up to present, no studies have been conducted for patients and parent’s perception about orthodontic treatment need in Bangladesh. Evaluation of patient’s perception of orthodontic treatment should be prioritized, as it is the patient who undergoes treatment and deserves satisfaction from improved aesthetics and function.^8^ Thus, the use of IOTN index provides potential to induce greater uniformity throughout the profession, along with standardization of assessment in orthodontic treatment, and facilitate better treatment procedures.^10^ Therefore, the present study was designed to detect the prevalence of malocclusion, related risk factors, and normative and perceived orthodontic treatment need in the population of children and adolescents in the city of Dhaka, Bangladesh; and also children’s and parent’s aesthetic perception, compared to orthodontist’s opinion, according to IOTN grades. 

## MATERIAL AND METHODS

The study took place from January 2014 to July 2014, and was approved by an ethical committee (ref. 2013/714), which complies with the Declaration of Helsinki. Four schools were randomly selected among all junior-high schools in the city of Dhaka, Bangladesh, and a random sample of 1,200 children aged 11-15 years attending these schools was selected. The questionnaire was translated into their mother tongue (Bengali), for better understanding. Parent’s questionnaire sheet included informed written consent, a questionnaire related to sociodemographic aspects (sex, age, employment status, education level), questions about dental and orthodontic history, as well as factors related to malocclusions (occurrence and duration of breastfeeding, non-nutritive sucking habits such as finger and pacifier, experience of caries and or extractions of deciduous teeth for any reason), and concern for orthodontic treatment need for their children. Parents also scored the AC of the IOTN of their children. For this purpose, ten photographs were sent to the parents, and detailed written instruction was given to identify the dental appearance that mostly resembled that of their child. A total of 800 parents returned their copies.

Prior to dental examination, trained and standardized personnel interviewed all children on previous experience about dental services, use of orthodontic devices and concern for the orthodontic treatment need. The AC of IOTN was also given to children, and each student was asked to identify which photograph of the AC scale most closely resembled the appearance of their anterior teeth. Children wearing orthodontic devices were also included in the descriptive analysis.

The examinations were carried out at school in broad daylight, by one trained and calibrated dentist, with the patient seated on a chair and using a portable equipment with mirrors, probe and ruler; no radiographs were taken. The Decayed, Missing, Filled Teeth (DMFT) index, according to WHO diagnostic criteria, was used to record caries experience^15^. The DHC and the AC of IOTN were calculated, and the Angle classification of malocclusions was recorded, with teeth in centric occlusion. The grading was done according to ‘Dental Health Component’ and ‘Aesthetic Component’ originally used in the study for development of the Index of Orthodontic Treatment Need.^8^ The five grades for DHC were: Grade 1) No need for orthodontic treatment; Grade 2) Little need for orthodontic treatment; Grade 3) Borderline need for orthodontic treatment; Grade 4) Great need for orthodontic treatment; Grade 5) Very great need for orthodontic treatment. The most severe occlusal trait was identified by the examiner and used to classify the patient, with scores ranging from 1 to 5. The aesthetic component (AC) was also recorded for each participant. Each participant’s anterior teeth were evaluated and a recording was made according to the photograph that most closely resembled the state of their dental appearance. The grades of the photographs indicated three treatment categories: Grade 1-4) No or little need of treatment; Grade 5-7) Borderline need of treatment; Grade 8-10) Definite need for treatment.

The definition of the questionnaire to reveal major difficulties and weaknesses was performed by means of a pilot study, using a convenience sample of children and parents. 

### Sample size calculation

The following formula was used to calculate the sample size (SS).

$$SS\;=\frac{\left[{\left(Z\alpha \;+\;Z\beta \right)}^{2}x\;\left(p\right)\;x\;\left(1-p\right)\right]}{d^{2}}$$

Where:

» Zα = 1.96 Z value (e.g.: 1.96 for 95% confidence level);
» Zβ = 0.84 power 80%;
» p = percentage picking a choice, expressed as decimal (0.50 used for sample size needed);
» d = confidence interval, expressed as decimal (e.g.: 0.05 = ±5);
» SS (Sample Size)= 784;
» Respondent non-response error = 784 x 0.05 = 39.2;
» Total sample size (784+39) = 823;

However, 800 sample size was taken for the study purposes. 

### Statistical analysis

All data were analyzed by means of standard statistical methods, using SPSS version 20, IBM Corp., USA. Descriptive analysis was carried out for the presentation of the results. Proportion test was used to compare definite treatment need (Grade 4-5) among boys and girls, and comparison between clinician, children and parent’s perceived AC scores. 

Three models were developed using stepwise multiple logistic regression analyses, in children who did not use orthodontic appliances: overall orthodontic treatment need according to IOTN (DHC Grade 4-5) (Model 1), orthodontic treatment need perceived by parents (Model 2) and by children (Model 3). The following variables were included in all models: age (continuous); gender (0 = male; 1 = female); highest education level of father (0 = none; 1 = elementary school; 2 = junior high school; 3 = high school; 4 = university); breastfeeding (0 = no; 1 = < 6 months; 2 = 6-12 months; 3 = > 12 months); non-nutritive sucking habits (0 = never; 1 = 1-12 months; 2 = 13-24 months; 3 = 25-36 months; 4 = 37-48 months; 5 = > 48 months); reported caries in deciduous teeth (0 = no; 1 = yes); reported extractions of deciduous teeth (0 = no; 1 = yes), and DMFT index (continuous). In models 2 and 3, the following variables were also included: orthodontic treatment need for overjet (0 = no; 1 = yes); crowding/spacing (0 = no; 1 = yes), and crossbite (0 = no; 1 = yes). Adjusted odds ratio (OR) and 95% confidence intervals (CI) were calculated. Logistic regression analysis was used to identify the factors associated to normative and perceived orthodontic treatment need.

Examiner reliability was checked in the pilot study by means of intra-examiner replicate examinations for 20 subjects, at a time interval of 20 days, and evaluated by the Kappa score. Reproducibility of the AC score was recorded also for the evaluation of a group of 20 children and 20 parents, by means of the Kappa score. 

## RESULTS

A total number of 800 schoolchildren, aged between 11 and 15 years, participated in this study. The sociodemographic and dental characteristics of the study population are reported in Table 1. 

**Table 1:** Selected characteristics of the study population.

Variables	n	%	Mean ± SD
Age			13.0 ± 1.4
Sex			
Male	403	50.4	
Female	397	49.6	
Breastfeeding			
No	2	0.3	
Yes	798	99.7	
Non-nutritive sucking habits			
No	624	78.0	
Yes	176	22	
Reported caries in the deciduous teeth			
Yes	315	39.4	
No	485	60.6	
Reported extraction of deciduous teeth for any reason			
Yes	136	17.0	
No	664	83.0	
Orthodontic devices need according to parents			
Yes	147	18.4	
No	647	80.8	
Using	6	0.8	
Orthodontic devices need according to children			
Yes	178	22.2	
No	616	77.0	
Using	6	0.8	
Visited a dentist in the previous year	198	24.7	
DMFT ≥ 1	172	21.5	1.57 ± 0.88
DMFT=0	628	78.5	

*DMFT = decayed missing filled teeth.

About one third, i.e. 39.4%, had at least one dental caries lesion; 17% had a history of one extraction of deciduous teeth; 24.7% had visited a dentist in the previous year, and only 0.8% of the children had already used orthodontic devices. The mean DMFT score was 1.57, and the standard deviation was 0.88.

**Figure 1:**
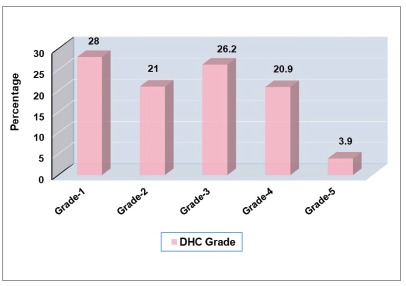
Percentage distribution of schoolchildren in the city of Dhaka, according to the DHC of the IOTN (Grade 1-2 = no or little need; Grade 3 = borderline need; Grade 4-5 = definite need).

The percentage distribution of the schoolchildren according to the DHC of the IOTN showed that almost half of them (49%) were in the category of ‘little or no treatment need’ (Grade 1-2), 26.2% were in the category of ‘borderline treatment need’ (Grade 3), and only 24.7% were in the category of ‘definite need’ (Grade 4-5) for orthodontic treatment (Fig 1). 

Table 2 shows the gender distribution of the treatment need according to the DHC grade of the IOTN: 23.3% of the total number of boys were in the category of ‘definite need’ (Grade 4-5), while 26.2% of the total number of girls were in this category; 27.8% of boys were in ‘borderline need’ (Grade 3) and 48.9% were in ‘no need’ (Grade 1-2) category. Meanwhile, ‘borderline need’ and ‘no need’ treatment for girls were 24.7% and 49.1%, respectively. Chi-square test showed that gender distribution of the DHC was not statistically significant [x^2^=4.09; *p*= 0.399]. A proportion test was used to compare ‘definite treatment need’ (Grade 4-5) among boys and girls. Total ‘definite treatment need’ (Grade 4-5) category was 24.7%, of which girls presented 26.2% - a slightly higher percentage than boys (23.3%). So, girls presented more treatment need than boys, although it was not statistically significant (*p*= 0.293).

**Table 2:** Assessment of the malocclusion severity and treatment need, among boys and girls, based on DHC of IOTN.

DHC of IOTN	Treatment need	Boys		Girls		Total		p-value
		n	%	n	%	n	%	
Grade 4-5	Definite need	94	23.3	104	26.2	198	24.7	0.293
Grade 3	Borderline need	112	27.8	98	24.7	210	26.3	0.319
Grade 1-2	Slight / No need	197	48.9	195	49.1	392	49.0	0.909
Total		403	100	397	100	800	100	

χ^2^ = 4.09; p=0.39.

**Figure 2:**
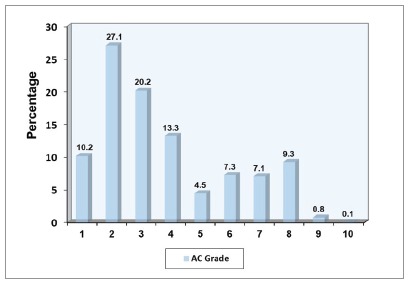
Percentage distribution of schoolchildren in the city of Dhaka according to the AC of the IOTN (Grade 1- 4 = slight or no need; Grade 5-7 = moderate need; Grade 8-10 = great need).

Figure 2 illustrates the distribution of ratings for the AC of IOTN in the school population: 70.8% of the children had ‘slight or no need’ (Grade 1-4) for orthodontic treatment, 19.0% had ‘moderate need’ (Grade 5-7), and 10.2% had ‘great need’ (Grade 8-10) for orthodontic treatment.

Table 3 shows the distribution of aesthetic self-perception of schoolchildren by gender, according to the AC of the IOTN. The highest distribution, 90% of the children, scored their teeth as aesthetically acceptable, indicating ‘no or little treatment need’ (Grade 1-4); while 5.4% and 4.6% of the children thought they best fitted into ‘borderline’ and ‘definite need treatment’ categories, respectively. Females graded themselves as being less attractive, and significantly desired more orthodontic treatment than males, according to Grade 8-10 (*p*= 0.0901).

**Table 3:** Distribution of aesthetic self-perception of schoolchildren by gender, according to AC of IOTN.

AC grade	Treatment need	Male		Female		Total		p-value
		n	%	n	%	n	%	
1-4	No/ Little need	356	92	325	87.8	681	90	0.054*
5-7	Borderline need	18	4.6	23	6.2	41	5.4	0.329
8-10	Definite need	13	3.4	22	6	35	4.6	0.090*
Total		387	100	370	100	757	100	

* Significant, p < 0.010.

It must be noted that 3.8% of the children could not be placed into any category of the AC of the IOTN, due to presenting Class III, open bite or anterior crossbite malocclusion (not included in AC of IOTN photographs). This did not adversely affect the statistical analysis of the data. 

Comparison between clinician, children and parents perceived AC scores (Table 4) shows that definite treatment need (AC score 8-10) was reported for 10.2% of subjects as assessed by the clinician, 4.6% by children, and 3.8% by parents. Borderline treatment need (AC score 5-7) was reported for 19.0% by clinician, 5.4% by children, and 6.2% by parents. Whereas most of the subjects were considered to have no need of orthodontic treatment (AC score 1-4): 70.8% by clinician, 90.0% by children, and 90.8% by parents. Clinician graded children as being in the less attractive side of the scale, compared to self-assessments made by children and parents. However, a proportion test showed that the differences between the clinician and children, and clinician and parents perceived AC score about the need for orthodontic treatment were highly statistically significant (*p*= 0.0001).

**Table 4:** Assessment of AC grades by clinician, children and parents.

AC grade	Need for treatment	Dentist		Child		Parent		Dentist vs Child	Dentist vs Parent
		n	%	n	%	n	%	p-value	p-value
1-4	No need	549	70.8	681	90.4	699	90.8	0.001	0.001
5-7	Borderline need	147	19.0	41	5.4	42	6.2	0.001	0.001
8-10	Definite need	79	10.2	35	4.6	29	3.8	0.001	0.001
Total		775	100	757	100	770	100		

* Significant, p < 0.001.

It must be noted that 3.1% by the clinician, 5.4% by the children and 3.8% by the parents could not be perceived in any category of the AC of the IOTN, due to presenting Class III, open bite or anterior crossbite malocclusion. This finding did not adversely affect the statistical analysis of the data.

Multiple logistic regression showed that dental health status was significantly related to orthodontic treatment need, since children with a higher DMFT were significantly more likely to need orthodontic treatment, according to the DHC of IOTN (OR = 1.77; 95% CI = 1.22-2.56; *p*= 0.002; Model 1 in Table 5). Orthodontic treatment need as perceived by parents and children was significantly predicted by treatment need for crowding/spacing (OR = 6.73; 95% CI = 1.93-2.34; *p*= 0.003; OR = 3.40; 95% CI = 1.36-8.50; *p*= 0.009) as measured by the IOTN (Models 2 and 3 in Table 5). However, caries experience, prolonged non-nutritive sucking habits and level of education of parents did not significantly affect the children self-perceived orthodontic treatment need (Model 3 in Table 5).

**Table 5:** Multivariable logistic regression models to examine the factors of overall orthodontic treatment need.

Variables	OR	SE	95% CI	P-value
Model 1: Orthodontic treatment need according to IOTN (DHC- 4-5) Log-likelihood= - 441.76; chi-square=11.79; P=0.008				
DMFT	1.77	0.33	1.22-2.56	0.002***
Breastfeeding	0.84	0.10	0.65-1.07	0.163
Gender	1.18	0.19	0.85-1.63	0.328
Model 2: Orthodontic treatment need perceived by parents Log-likelihood= - 88.16; chi-square=15.07; P=0.004				
Treatment need for crowding/spacing	6.73	4.28	1.93-23.4	0.003**
Age	0.78	0.11	0.59-1.03	0.086
Gender	1.03	0.40	0.48-2.19	0.942
Level of education of the parents	1.14	0.24	0.75-1.73	0.536
Model 3: Orthodontic treatment need perceived by children Log-likelihood= - 100.79; chi-square=21.23; P=0.001				
Treatment need for crowding/spacing	3.40	1.59	1.36-8.50	0.009*
Level of education of the parents	1.36	0.29	0.90-2.06	0.138
Prolonged non-nutritive sucking habits	1.26	0.18	0.94-1.67	0.111
Age	0.69	0.09	0.53-0.90	0.008*
DMFT	1.48	0.58	0.68-3.20	0.319
Gender	1.58	0.56	0.77-3.19	0.205

***P<0.001; **P<0.05; *P<0.010; *Significant.

DMFT= decayed missing filled teeth, DHC= dental health component.

### Reliability of the IOTN index

 The intra-examiner agreement for DHC showed a Kappa value of 0.875, indicating ‘high agreement’ between first and second readings; and for AC showed a Kappa value of 0.763, indicating ‘substantial agreement’ between first and second readings. However, children and parents AC scores showed moderate agreement, with Kappa scores of 0.331 and 0.565, respectively.

## DISCUSSION

Bangladesh is a South-East Asian country with a population of approximately 140 million people and an area of about 147,000 square kilometers. A large concentration of people lives in Dhaka, the capital and main city of Bangladesh. Bangladeshi demographic characteristic shows that this situation is very similar to that of other developing countries. In Bangladesh, there are limited postgraduate institutes for dental students, which are mostly situated in the city of Dhaka, among which only one is a public institute, the Dhaka Dental College, and provides orthodontic treatment with lower cost. In this context, patients who are referred to this public institute are treated based on severity of the malocclusion.

Thus, the primary goal of this survey was to detect the prevalence of malocclusions needing orthodontic treatment, in the population of children and adolescents in Bangladesh, and also the orthodontic treatment need as perceived by parents and children. Several published studies have already described the prevalence and types of malocclusions in different populations. Comparisons of these findings should be made with caution, since different methods and indices were used. 

The most interesting finding in the present study is that 49% of the total schoolchildren in the city of Dhaka were in the category of little or no treatment need (Grade 1-2), and only 24.7% were in the category of definite need (Grade 4-5) for orthodontic treatment, according to DHC of IOTN. The distribution of DHC and AC grades has been studied by several researchers. Comparing the studies that used DHC only, the frequency of definite treatment need (Grade 4-5) found in the present population (24.7%) was similar to the results found in Germany^4^ (26.2%), Norway^12^ (26.1%), Tanzania^16^ (22%), India^17^ (23.5%), and England^18^ (26.2%); but lower than those reported for Italy^1^ (59.5%), Turkey^9^ (38.8%), Peru^19^ (29.9%), Jordan^20^ (34%), Malaysia^21^ (53.9%), Japan^22^ (40%) and Hong Kong^23^ (54.2%); and higher than those reported for Iran^11^(18.41%), Finland^24^ (15%), Spain^25^ (17.1% and 21.8%), and France^26^ (21.3%).

The distribution with respect to male and female of orthodontic treatment need has also been studied by several researchers. In the present study, Chi-square test showed that gender distribution of the DHC was not statistically significant (χ^2^ = 4.09; *p*= 0.399). This finding was supported by other studies.^9^ ^,^ ^19^ ^,^ ^25^ ^-^ ^27^ When a proportion test was used to compare definite treatment need according to the DHC (Grade 4-5) among boys and girls, the total definite treatment need was 24.7%. Girls presented 26.2%, a slightly higher treatment need than boys (23.3%), although these results were not considered statistically significant (*p*= 0.293). These findings were supported by some studies,^26^ ^,^ ^27^ and at the same time opposed by others that reported that boys presented significantly more treatment need than girls.^11^ The differences in age group, ethnic background, geographical location, and level of expectations of each population may have contributed to the controversy.

According to the AC of the IOTN assessed by clinicians, orthodontic treatment need results were dramatically different, as the ‘no or little need’ for treatment (Grade 1-4) was 70.8%, and ‘definite need for treatment’ (Grade 8-10) was only 10.2%. In this study the frequency of ‘definite treatment need’ (Grade 8-10) was close to that of Norway^12^ (9%), Tanzania^16^(11%) and India^17^ (12.9%); but lower than the reported for Germany^4^ (21.5%), Jordan^6^ (21%) and Malaysia^21^ (46.7%); and higher than the reported for Italy^1^ (3.2%), Turkey^9^(4.8%), Iran^11^ (6.2%) and England^27^ (6%).

In contrast, when the students were asked to score their own dental appearance, according to the AC of the IOTN, the highest distribution (90%) of the children scored their teeth as aesthetically acceptable, indicating ‘no or little treatment need’ (Grade 1-4). While 5.4% and 4.6% of the children thought they best fitted in ‘borderline’ and ‘definite treatment need’ categories, respectively. A slightly greater number of females graded themselves at the least attractive scale, compared to males. This findings is supported by another study^12^ and opposed by other studies.^5^ ^,^ ^10^ However, they didn’t find any significant relationship between gender, which was not supported by the present study. Females desired significantly more orthodontic treatment than males, according to Grade 8-10 (*p*= 0.0901). This finding was supported by a study,^28^ but opposed by other study reporting that females significantly tended to rate their dentition as being more attractive than males.^20^ It is possible that the fact that more girls were concerned about minor deviations reflects a reduction in their self-esteem.

Comparisons between clinician, children and parents perceived AC scores showed that clinician graded children to the less attractive side of the scale, compared to the self-assessments made by children and parents. The clinician allocated more subjects to the ‘borderline’ and ‘definite need’ categories (29.2%) than did children and parents. A proportion test showed that differences between the clinician and children, and clinician and parents perceived AC score were highly statistically significant (*p*= 0.0001). This result was supported by a study.^1^ Some other studies (parents were not included) showed that clinicians’ perceived treatment need was more critical than childrens’, and found significant differences between clinician and patients perceived score,^5^ ^,^ ^12^ ^,^ ^20^ which is in line with the present study. Another study found a significant relation only between clinician and children, which is corroborated by the present study; but the relation between clinician and parents perceived score was not significant, which contradicts the present study.^6^ Some other studies found no significant relation between clinician and children perceived AC score,^10^ ^,^ ^27^ which contradicts the present study. The different age groups and different ethnic backgrounds may have contributed to this controversy. It is possible that subjects replied defensively and subconsciously, trying to allocate themselves to the attractive side, in order to avoid treatment. Alternatively, as photographs showed dentition only in the frontal view, it is possible that the patients did not perceive their own dentition in the AC scale, in cases of anterior crossbite, Class III, open bite, spaces due to unerupted canine or missing lateral incisors. 

The results of the multivariate analysis showed that the only determinant of normative orthodontic treatment need, according to the DHC, was high DMFT scores. This finding was supported by other studies^1^ ^,^ ^3^. The positive finding of this study was that orthodontic treatment need as perceived by parents and children was significantly predicted by treatment need for crowding and spacing, as measured by the IOTN. However, caries experiences, prolonged non-nutritive sucking habits and the level of education of parents did not significantly affect children’s self-perceived orthodontic treatment need. This finding was supported by other studies^1^ ^,^ ^11^. Subjective perception of orthodontic treatment need was significantly associated, for both parents and children, to treatment need for crowding and spacing, which is not surprising, since these are the most ‘visible’ types of malocclusions. These findings emphasize the need to educate the population to recognize other kinds of malocclusions.

### Limitations of the study

The present study have limitations such as: the subjects were selected from schoolchildren and adolescents only from the city of Dhaka, Bangladesh. A large population from different regions of the country and socio-economic status may present an influence over the perception and concern of orthodontic treatment needs. Another limitation in this study was that parents’ questionnaires were sent through their child, instead of arranging interview with parents together with their child, on dental examination day. This may be the reason why some parents did not return their copies, due to lack of interest in participating in the study, which may have influenced the results. It was noticeable that assessment of IOTN-AC required considerable effort for understanding by adolescents and parents, due to the lack of some categories of the AC of the IOTN, such as Class III, open bite, and anterior crossbite malocclusion. Nevertheless, the authors are confident that the detailed instructions provided, along with intra-examiner reliability test performed in the pilot study, create great evidence to consider this study valid and reliable. 

## CONCLUSION

Based on the present results, the following conclusion can be drawn:

» A low proportion of schoolchildren presented definite normative orthodontic treatment need. One-fourth of schoolchildren (24.7%) had definite treatment need (Grade 4-5), and 10.2% presented great need (Grade 8-10), based on DHC and AC of the IOTN, respectively, and should be prioritized for orthodontic services.
» The only determinant of normative treatment need was DMFT, although it was not associated with perceived orthodontic treatment.
» Regarding aesthetics, clinician’s perceived the children as being less attractive than the self-assessment of children and parents, according to the AC of IOTN.
» Females graded themselves as less attractive than males, and significantly desired orthodontic treatment more than males, according to the AC Grade 8-10.

## References

[1] Nobile CG, Pavia M, Fortunato L, Angelillo IF (2007). Prevalence and factors related to malocclusion and orthodontic treatment need in children and adolescents in Italy. Eur J Public Health.

[2] Shivakumar KM, Chandu GN, Reddy VVS, Shafiulla MD (2009). Prevalence of malocclusion and orthodontic treatment needs among middle and high school children of Davangere city, India by using Dental Aesthetic Index. J Indian Soc Pedod Prev Dent.

[3] Mtaya M, Brudvik P, Astrom AN (2009). Prevalence of malocclusion and its relationship with sociodemographic factors, dental caries, and oral hygiene in 12 to 14 year old Tanzanian schoolchildren. Eur J Orthod.

[4] Tausche E, Luck O, Harzer W (2004). Prevalence of malocclusion in the early mixed dentition and orthodontic treatment need. Eur J Orthod.

[5] Aikins EA, Dacosta OO, Onyeaso CO, Isiekwe MC (2012). Self-perception of malocclusion among Nigerian adolescents using the aesthetic component of the IOTN. Open Dent J.

[6] Hamdan AM (2004). The relationship between patient, parent and clinician perceived need and normative orthodontic treatment need. Eur J Orthod.

[7] Hamdan AM, Al-Omari IK, Al-Bitar ZB (2007). Ranking dental aesthetics and thresholds of treatment need a comparison between patients, parents, and dentist. Eur J Orthod.

[8] Brook PH (1989). And Shaw WC The development of an index of orthodontic treatment. Eur J Orthod.

[9] Ucuncu N, Ertugay E (2001). The use of the Index of Orthodontic Treatment Need (IOTN) in a school population and referred population. J Orthod.

[10] Sharma J, Dhir RS (2014). IOTN-A tool to prioritize treatment need in children and plan dental health services. Oral Health Dent Manag.

[11] Hedayati Z, Fattahi HR, Jahromi SB (2007). The use of orthodontic treatment need in an Iranian population. J Indian Soc Pedod Prev Dent.

[12] Birkeland K, Boe OE, Wisth PJ (1996). Orthodontic concern among 11 -year- old children and their parents compared with orthodontic treatment need assessed by index of orthodontic treatment need. Am J Orthod Dentofacial Orthop.

[13] Chew MT, Aw AKL (2002). Appropriateness of orthodontic referrals self-perceived and normative treatment needs of patients referred for orthodontic consultation. Community Dent Oral Epidemiol.

[14] Islam MR (2009). The need for orthodontic treatment among Bangladeshi school children in late mixed dentition stage. Bangladesh Dent J.

[15] World Health Organization (1987). Oral Health Surveys. Basic Methods.

[16] Mugonzibwa EA, Kuijpers-Jagtman AM, Vant Hof MA, Kikwilu EN (2004). Perceptions of dental attractiveness and orthodontic treatment need among Tanzanian children. Am J Orthod Dentofacial Orthop.

[17] Diwan S, Kumar S, Saxena V, Goel D (2013). Assessment of orthodontic treatment needs among children in Doiwala region, Uttarakhand, India. Natl J Community Med.

[18] Tickle M, Kay EJ, Bearn D (1999). Socio-economic status and orthodontic treatment need. Community Dent Oral Epidemiol.

[19] Bernabe E, Mir CF (2006). Normative and self-perceived orthodontic treatment need of a Peruvian University population. Head Face Med.

[20] Abu Alhaija ESJ, Al-Nimri KS, Al-Khateeb SN (2004). Orthodontic treatment need and demand in 12-14-year-old north Jordanian school children. Eur J Orthod.

[21] Zreaqat M, Hassan R, Ismail AR, Ismail NM, Aziz FA (2013). Orthodontic treatment need and demand among 12- and 16 year-old school children in Malaysia. Oral Health Dent Manag.

[22] Ekuni D, Furuta M, Irie K, Azuma T, Tomofuji T, Murakami T (2011). Relationship between impacts attributed to malocclusion and psychological stress in young Japanese adults. Eur J Orthod.

[23] Tang EL, So LL (1995). Correlation of orthodontic treatment demand with treatment need assessed using two indices. Angle Orthod.

[24] Kerosuo H, Kerosuo E, Niemi M, Simola H (2000). The need for treatment and satisfaction with dental appearance among young Finnish adults with and without a history of orthodontic treatment. J Orofac Orthop.

[25] Manzanera D, Montiel-company JM, Almerich-Silla JM, Gandia JL (2009). Orthodontic treatment need in Spanish schoolchildren an epidemiological study using the Index of Orthodontic Treatment Need. Eur J Orthod.

[26] Souames M, Bassigny F, Zenati N, Riordan PJ, Boy-Lefevre ML (2006). Orthodontic treatment need in French schoolchildren an epidemiological study using the Index of Orthodontic Treatment Need. Eur J Orthod.

[27] Mandall NA, Mccord JF, Blinkhorn AS, Worthington HV, O'Brien KD (2000). Perceived aesthetic impact of malocclusion and oral self-perception in 14-15 year old Asian and Caucasian children in Greater Manchester. Eur J Orthod.

[28] Alsaker FD, Olweus D (1993). Global self-evaluations and perceived instability of self in early adolescence a cohort longitudinal study. Scand J Psychol.

